# Morphine Enhances HIV-1_SF162_-Mediated Neuron Death and Delays Recovery of Injured Neurites

**DOI:** 10.1371/journal.pone.0100196

**Published:** 2014-06-20

**Authors:** Ruturaj R. Masvekar, Nazira El-Hage, Kurt F. Hauser, Pamela E. Knapp

**Affiliations:** 1 Department of Anatomy and Neurobiology, Virginia Commonwealth University, Richmond, Virginia, United States of America; 2 Department of Pharmacology and Toxicology, Virginia Commonwealth University, Richmond, Virginia, United States of America; 3 Institute for Drug and Alcohol Studies, Virginia Commonwealth University, Richmond, Virginia, United States of America; University of Nebraska Medical Center, United States of America

## Abstract

HIV-1 enters the CNS soon after initial systemic infection; within the CNS parenchyma infected and/or activated perivascular macrophages, microglia and astrocytes release viral and cellular toxins that drive secondary toxicity in neurons and other cell types. Our previous work has largely modeled HIV-neuropathology using the individual viral proteins Tat or gp120, with murine striatal neurons as targets. To model disease processes more closely, the current study uses supernatant from HIV-1-infected cells. Supernatant from HIV-1_SF162_-infected differentiated-U937 cells (HIV^+^_sup_) was collected and p24 level was measured by ELISA to assess the infection. Injection drug abuse is a significant risk factor for HIV-infection, and opiate drug abusers show increased HIV-neuropathology, even with anti-retroviral treatments. We therefore assessed HIV^+^_sup_ effects on neuronal survival and neurite growth/pruning with or without concurrent exposure to morphine, an opiate that preferentially acts through µ-opioid receptors. Effects of HIV^+^_sup_ ± morphine were assessed on neuronal populations, and also by time-lapse imaging of individual cells. HIV^+^_sup_ caused dose-dependent toxicity over a range of p24 levels (10–500 pg/ml). Significant interactions occurred with morphine at lower p24 levels (10 and 25 pg/ml), and GSK3β was implicated as a point of convergence. In the presence of glia, selective neurotoxic measures were significantly enhanced and interactions with morphine were also augmented, perhaps related to a decreased level of BDNF. Importantly, the arrest of neurite growth that occurred with exposure to HIV^+^_sup_ was reversible unless neurons were continuously exposed to morphine. Thus, while reducing HIV-infection levels may be protective, ongoing exposure to opiates may limit recovery. Opiate interactions observed in this HIV-infective environment were similar, though not entirely concordant, with Tat/gp120 interactions reported previously, suggesting unique interactions with virions or other viral or cellular proteins released by infected and/or activated cells.

## Introduction

Human immunodeficiency virus-1 (HIV-1) disrupts normal immune system function and leads to acquired immunodeficiency syndrome (AIDS). HIV-1 can also induce a wide range of central nervous system (CNS) deficits, collectively known as HIV-1-associated neurocognitive disorders (HAND). HIV-1 enters the CNS soon after initial systemic infection [1], [2]. It is widely believed that virus penetrates the CNS within infected monocytes and lymphocytes [2], [3], which normally traffic across the blood-brain barrier (BBB) as a part of immune surveillance of the brain. Mature neurons are not infected by HIV-1; instead, infected and/or activated glial cells release various viral and cellular factors that induce direct and/or indirect neuronal toxicity, leading to HAND [2], [4]–[7]. Combination antiretroviral therapy (cART), which controls systemic HIV-infection, has improved the health status of a large segment of patients [8]–[10]. Although cART has reduced the overall severity of neurocognitive disorders in HIV-1 patients, the prevalence of HAND remains at approximately 50% [4], [8], [10]–[12]. The persistence of relatively high rates of CNS disease is likely due to a combination of longer patient survival, the relatively poor CNS penetrance of most antiretroviral drugs [4], [13], and their neurotoxic effects [14]. Even if the CNS viral load is extremely low or undetectable, neurodegeneration can still occur in response to viral proteins, such as transactivator of transcription (Tat), that are released from cells even when viral replication has been inhibited [15].

Injection drug abusers are at high risk of acquiring HIV-infection due to sharing of contaminated needles and unsafe sexual behavior. Nearly 30% of HIV-infected patients have a history of injection drug abuse involving opiates [16], [17]. Additionally, a subset of HIV^+^ patients is exposed to opiates through their legitimate use for treatment of AIDS-related chronic pain syndromes. As opiates by themselves are known to induce immunomodulatory or immunosuppressive effects, both in the periphery and CNS [18], [19], it is hypothesized that they may enhance virus spread or otherwise exacerbate disease processes. Experimental evidence also suggests that opiates can interact with HIV-1 or HIV-1-proteins directly on CNS cells and tissues [16], [20]–[26]. Among patients with HIV-1 infection, those who also abuse opiate drugs show faster progression to AIDS and more severe neurocognitive deficits [27]–[29].

Many previous studies have modeled HIV-neuropathology using individual viral proteins, such as Tat, glycoprotein 120 (gp120), and others. However, the CNS of HIV-infected patients is not only exposed to individual viral proteins, but instead to all cytokines/chemokines and other cellular products, viral proteins and virus particles released from infected and/or activated cells. Thus, to more closely model HIV-1-mediated neurotoxicity, we have used supernatant from HIV-1_SF162_-infected differentiated-U937 cells (HIV^+^_sup_). The R5-tropic HIV-1_SF162_ strain was used since R5-tropic (monocyte/macrophage-tropic) viruses are predominant in cerebrospinal fluid (CSF) and CNS parenchyma [30], [31].

Multiple outcome measures were studied after HIV^+^_sup_ ± morphine treatments, including both cell death and neurite degeneration. Studies in the presence of glia allowed us to distinguish between direct and indirect neurotoxicity. Our results indicate that morphine worsens selective neurotoxic effects of HIV, and that glycogen synthase kinase-3β (GSK3β) signaling may be a point of convergence. Importantly, morphine limits the ability of neurons to recover from sublethal damage.

## Material and Methods

### Ethics statement

Experiments were conducted in accordance with procedures reviewed and approved by the Virginia Commonwealth University Institutional Animal Care and Use Committee (Protocol Number: AM10158).

### Mixed glial cultures

Cells were cultured from the striatum, a region targeted by HIV where opioid receptor levels are relatively high. Mixed glial cultures (astrocytes and microglia) from mouse striatum were prepared as previously described [23], [24], with minor modifications. Striata from P0-P1 ICR (CD-1) mice (Charles River Laboratories International, Inc., Wilmington, MA) were dissected, minced and enzymatically dissociated with trypsin (2.5 mg/ml; Sigma-Aldrich, St. Louis, MO) and deoxyribonuclease (DNase; 0.015 mg/ml; Sigma-Aldrich) in Dulbecco's Modified Eagle's Medium (DMEM; Gibco, Grand Island, NY) for 30 min at 37°C. Tissue was resuspended in DMEM supplemented with 10% fetal bovine serum (FBS; Gibco), triturated and filtered through 100 and 40 µm nylon mesh pore filters respectively. Cells were plated and maintained in supplemented DMEM containing 10% FBS.

### Neuronal cultures

Mouse striatal neuron cultures were prepared as previously described [23], [24]. Briefly, striata from E15-E16 ICR mice were dissected, minced and enzymatically dissociated with trypsin (2.5 mg/ml) and DNase (0.015 mg/ml) in neurobasal medium (Gibco) for 30 min at 37°C. Tissue was resuspended in neurobasal medium supplemented with B-27 additives (Gibco), L-glutamine (0.5 mM; Gibco) and glutamate (25 µM; Sigma-Aldrich), triturated and filtered twice through 70 µm nylon mesh pore filters. Neurons were plated and maintained in supplemented neurobasal medium. Culture purity was determined by immunocytochemistry using anti-MAP-2 antibody (Abcam, Cambridge, MA; ab32454) and found to be >80% neurons.

### Neuron-mixed glial co-cultures

All cultures were prepared in 24 well plates pre-coated with poly-L-lysine (0.5 mg/ml; Sigma-Aldrich). Neurons were plated alone in 12 wells (neuron cultures); in the remaining 12 wells we established neuron-glia co-cultures as previously described [23]. Briefly, two deep midline grooves were made into the culture surface to restrict the movement of glial cells between sides. Glial cells (2×10^5^ cells/well) were plated on one side of the grooves; when they became confluent (10 d), neurons (0.25×10^5^ cells/well) were plated onto the entire culture surface. In these wells, all neurons are exposed to glial conditioned medium, but neurons on one side of the grooves contact the glial bedlayer, while neurons on the other side grow in isolation on the culture surface. It is difficult to visualize all neurite extensions when neurons are in contact with the glial bedlayer. Thus, the co-culture studies used neurons that did not have direct contact with glia. All cultures were maintained in supplemented neurobasal medium; neurons were allowed to mature for 5–7 d prior to treatment.

### Supernatant from HIV-infected cells

U937 cells (ATCC, Manassas, VA), a leukemic monocyte cell line originally derived from a histiocytic lymphoma, were plated at 0.5×10^5^ cells/ml in RPMI-1640 media (Gibco) supplemented with 10% FBS, and activated/differentiated with interleukin-2 (IL-2, 100 ng/ml; Sigma-Aldrich), phytohaemagglutinin (PHA, 5 µg/ml; Sigma-Aldrich), and phorbol 12-myristate 13-acetate (PMA, 100 ng/ml; Sigma-Aldrich), for 48 h. Activated/differentiated cells were treated with Polybrene (2 µg/ml; Sigma-Aldrich) for 30 min at 37°C, and exposed to HIV-1_SF162_ (p24 = 50–100 pg/ml; from Dr. Jay Levy [32], through the NIH AIDS Research and Reference Reagent Program, Germantown, MD). After 7 d, supernatants were collected by filtering through a 0.20 µm filter. HIV infection was confirmed by quantification of p24 levels (HIV-1 p24 Antigen Capture Assay; Advanced Bioscience Laboratories, Rockville, MD) in culture supernatants; a 4-6 fold increase in p24 antigen levels was typical over 7 d. Supernatants from uninfected but differentiated U937 cells (Control_sup_) were used as a control. Cell culture supernatants were aliquoted and stored at −80°C.

### Treatments

Morphine is the major metabolite of heroin in the CNS [33]; it preferentially targets µ-opioid receptors (MORs). Since opiates by themselves can affect HIV-1 infection and replication [34], it was important to assess the effects of opiate interactions using cell-free supernatants. HIV^+^_sup_ or Control_sup_ were added to neuronal cultures in the presence or absence of morphine sulfate (500 nM; Sigma-Aldrich) ± naloxone (1.5 µM; Sigma-Aldrich), a general opioid receptor antagonist.

### MTT assay

At specific times after treatment, cells were rinsed and incubated with 1.2 mM 3-(4,5-Dimethylthiazol-2-yl)-2,5-diphenyltetrazolium bromide (MTT; Molecular Probes, Grand Island, NY) in fresh, pre-warmed media at 37°C for 4 h. The medium was gently aspirated, and formazan crystals, the product of reduction of MTT by mitochondrial dehydrogenase in live cells, were dissolved in 100 µl of dimethyl sulfoxide (DMSO; Sigma-Aldrich) at 37°C for 10 min. The amount of formazan was measured by absorbance at 540 nm using a PHERAstar microplate reader (BMG LABTECH Inc., Cary, NC).

### TUNEL assay

At specific intervals after treatments, cells were fixed overnight at 2–8°C in 4% paraformaldehyde (Sigma-Aldrich), permeabilized at room temperature in 0.1% Triton-X 100 (Molecular Probes) and 0.1% BSA (Invitrogen, Grand Island, NY) for 15 min, and blocked in 0.1% BSA and 1% horse serum for 30 min. Fixed cells were stained for Hoechst 33342 (Sigma-Aldrich) and terminal deoxynucleotidyl transferase dUTP nick end labeling (TUNEL; Roche Applied Sciences, Mannheim, Germany). Cells were visualized and digital images were acquired using an Axio Observer Z.1 microscope and Zen 2010 software (Zeiss Inc., Thornwood, NY). Neuronal apoptosis was assessed by manually counting the percentage of TUNEL(+) cells.

### Assessment of neuronal viability

In each culture well, at least 50 healthy neurons were initially selected in 6–8 non-overlapping fields. After treatment, repeated images of pre-selected cells were captured at 1 h intervals, using a microscope with a computer-regulated stage (Axio Vision 4.6; Carl Zeiss Inc.) under controlled environmental conditions (37°C, 95% humidity and 5% CO_2_) [23], [24]. At the end of each experiment, pre-selected neurons were assessed for viability at 6 h intervals in the digital images. Cell death was confirmed using rigorous morphological criteria including abnormal shrinking of the cell body and eventual cell body fragmentation, nuclear destruction, loss of phase-brightness, and excessive neurite loss [23], [24], [35]. In some experiments, live and dead cells were confirmed at the end of the experiment by staining respectively with calcein-AM and ethidium homodimer-1 (LIVE/DEAD Viability/Cytotoxicity Kit; Molecular Probes, Grand Island, NY). Findings were reported as the average percentage of neuron survival, with respect to pre-treatment neuron count ± standard error of the mean (SEM), and analyzed using a repeated measure analysis of variance (ANOVA) and Duncan's post hoc test using Statistica 8.0 (StatSoft, Tulsa, OK).

### Assessment of neurite length

At specific intervals after treatments, cells were fixed, permeabilized, blocked, and subsequently stained for TUNEL and MAP-2 (Abcam; ab32454); cells were visualized and digital images were acquired. In digital images, neuritic arborization was quantified only for live [TUNEL(-)] neurons, using modified Sholl analysis. A ‘Sholl score’ was measured by counting the number of intersections of MAP-2-positive neurites with equidistant concentric circles of increasing radius, centered on the cell body [36]. The Sholl score was converted into neurite length in µm via micrometer calibration at the same magnification.

### Assessment of neurite growth/regrowth after treatment removal

Prior to treatment, at least 15 healthy neurons were selected in 7–8 non-overlapping fields per well. Repeated images of pre-selected cells were captured at 1 h intervals after treatment onset. After 24 h, cells were gently rinsed with pre-warmed medium and returned either to a ‘control ± opiate environment’, which had Control_sup_, or to a ‘HIV ± opiate environment’ which had HIV^+^_sup_ (Table 1). We then continued to capture images of the same neurons at 1 h intervals for an additional 48 h (total 72 h). Thus, there were a total of 9 groups with varying exposure times to HIV^+^_sup_ or Control_sup_, in the presence or absence of morphine ± naloxone; these are outlined in Table 1.

**Table 1:** Treatment paradigm for neurite growth/regrowth assessment.

Treatment Groups	Treatment from 0 to 24 h	Treatment from 24 to 72 h
**72 h (C)**	Control	Control
**72 h (C+M)**	Control + Mor	Control + Mor
**72 h (C+M+N)**	Control + Mor + Nal	Control + Mor + Nal
**72 h (H)**	HIV	HIV
**72 h (H+M)**	HIV + Mor	HIV + Mor
**72 h (H+M+N)**	HIV + Mor + Nal	HIV + Mor + Nal
**24 h (H) then 48 h (C)**	HIV	Control
**24 h (H+M) them 48 h (C+M)**	HIV + Mor	Control + Mor
**24 h (H+M+N) then 48 h (C+M+N)**	HIV + Mor + Nal	Control + Mor + Nal

Control  =  Control_sup_; HIV  =  HIV^+^_sup_ (p24 = 25 pg/ml); Mor  =  morphine sulfate (500 nM); Nal  =  naloxone (1.5 µM).

Neurons that remained alive until the experiment end (72 h) were assessed for neuritic arborization in images taken at 0 h, 24 h, and 72 h using Sholl analysis. The findings were reported as average Sholl scores at each time, normalized to pre-treatment (0 h) scores ± SEM. Data were analyzed using a repeated measure ANOVA and Duncan's post hoc test using Statistica 8.0 (StatSoft).

### ELISA

Conditioned medium from mixed glial cultures were collected on the schedule described in Table 1 and assessed for brain-derived neurotrophic factor (BDNF), glial cell-derived neurotrophic factor (GDNF), interleukin 6 (IL-6) and tumor necrosis factor α (TNFα) by ELISA according to the manufacturer's instructions (BDNF and GDNF ELISAs: Abcam; IL-6 and TNFα ELISAs: R&D Systems, Minneapolis, MN). 3,3′,5,5′-tetramethylbenzidine (TMB) substrate was added for color development and plates were read at 450 nm using a PHERAstar microplate reader immediately after terminating the reaction. Protein levels were determined based on a standard curve.

### Immunoblotting

Whole cell extracts were prepared using radioimmunoprecipitation assay (RIPA) buffer (Sigma-Aldrich) with protease and phosphatase inhibitors (cOmplete - protease inhibitor cocktail tablets, and PhosSTOP - phosphatase inhibitor cocktail tablets; Roche), and total protein concentrations were determined by bicinchoninic acid (BCA) assay (Thermo Fisher Scientific, Rockford, IL). Cell lysates containing equal amounts of total protein (∼5–10 µg) were heated at 100°C for 5 minutes in laemmli buffer (Sigma-Aldrich), electrophoretically separated on a 10% SDS-polyacrylamide gels (Criterion Precast Gel; Bio-Rad, Hercules, CA), and transferred onto polyvinylidene difluoride (PVDF) membranes (Immun-Blot; Bio-Rad). Membranes were incubated with primary antibodies for phospho-GSK3β-Ser9 (p-GSK3β-S9; Cell Signaling Technology, Danvers, MA; 5558), GSK3β (t-GSK3β; Cell Signaling Technology; 9832) and glyceraldehyde 3-phosphate dehydrogenase (GAPDH; Abcam; ab8245). Appropriate horseradish peroxidase-conjugated secondary antibodies (SouthernBiotech, Birmingham, AL) were used. Membranes were detected using SuperSignal West Femto Maximum Sensitivity Substrate (Thermo Fisher Scientific), and visualized using a Kodak Image Station 440CF.

### Statistical analyses

All data were expressed as average ± SEM. Unless otherwise indicated, data were analyzed statistically using a one-way ANOVA followed by Duncan's post hoc test using Statistica 8.0 (StatSoft); an α level of *p*<0.05 was considered significant.

## Results

### Dose dependent neuron death and interactions with morphine

**Figure 1:**
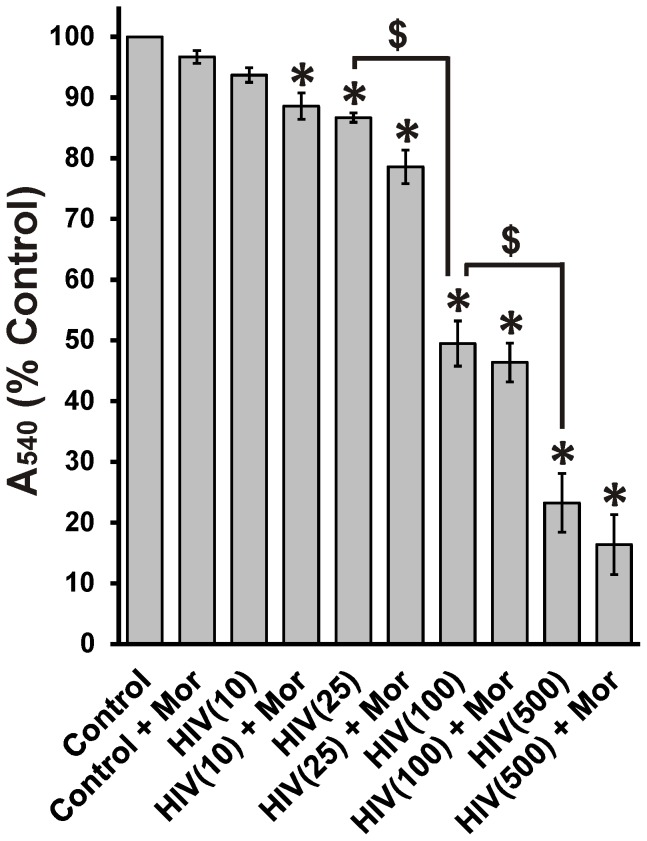
Concentration-dependent change in MTT reduction. Cell toxicity/proliferation was analyzed in neuron cultures at 48 h after treatment using an MTT assay. The findings were reported as percent of control absorbance at 540 nm (A_540_) ± SEM. Significance was analyzed using a one-way ANOVA and Duncan's post hoc test, from *n*  = 3 separate experiments. All treatment groups, except morphine alone [Control + Mor] and p24 = 10 pg/ml of HIV^+^_sup_ [HIV(10)], showed significantly decreased absorbance at 540 nm (*p<0.05 vs. Control), likely reflecting neurotoxicity. HIV^+^_sup_ caused a concentration-dependent reduction in A_540_ (^$^ *p*<0.05). Morphine did not show a significant interaction with HIV^+^_sup_ at any p24 level. Control  =  Control_sup_; HIV  =  HIV^+^_sup_ (concentration of p24 in pg/ml is specified in parentheses); Mor  =  morphine sulfate (500 nM).

**Figure 2:**
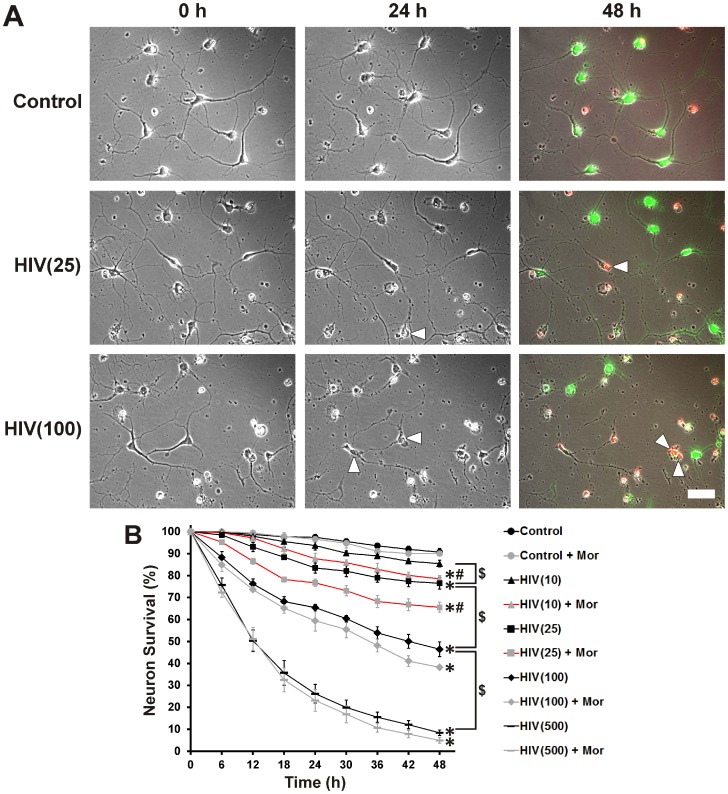
Concentration-dependent neuronal death in cultures treated with HIV^+^_sup_ ± morphine. Individual striatal neurons were selected prior to treatment and repeatedly imaged for 48(**A**) Digital images of the same cells/fields, at 0 h, 24 h and 48 h after treatment (white arrowheads indicate cells that have died over the previous 24 h period). Live and dead cells were confirmed at the end of the experiment by staining respectively with calcein-AM (green) and ethidium homodimer-1 (red); scale bar  = 40 µm. (**B**) Cells were assessed for viability at 6 h intervals in digital images. Findings were reported as the average neuronal survival as a percent of pre-treatment neuron count ± SEM. Significance was analyzed by repeated measures ANOVA and Duncan's post hoc test, from *n* = 3 separate experiments (at least 150 neurons per treatment group). Over the period of 48 h, all treatment groups except morphine alone [Control + Mor] and p24 = 10 pg/ml of HIV^+^_sup_ alone [HIV(10)], showed significantly reduced neuronal survival (*p<0.05 vs. Control). Neuronal survival declined in a concentration dependent manner with HIV^+^_sup_ treatment (^$^ *p*<0.05). Morphine showed significant interaction with HIV^+^_sup_, but only at lower levels of exposure (p24 = 10 and 25 pg/ml) (^#^ *p*<0.05 vs. HIV^+^_sup_ alone at corresponding titer). Control  =  Control_sup_; HIV  =  HIV^+^_sup_ (concentration of p24 in pg/ml is specified in parentheses); Mor  =  morphine sulfate (500 nM).

To determine the HIV^+^_sup_ concentration for subsequent experiments we assessed concentration-dependent toxicity in the presence or absence of 500 nM morphine, a titer chosen to maximally stimulate neuronal and glial MORs *in vitro*, and to result in dynamic Ca^2+^ changes and secretion of multiple cytokines and chemokines [21], [23]–[26], [37]–[39]. HIV^+^_sup_ at p24≥25 pg/ml and above showed significant toxicity even in the absence of morphine in an MTT assay. As expected, there was a concentration-dependent decrease in MTT reduction, indicating lower mitochondrial activity at higher HIV^+^_sup_ levels. At p24 concentrations ≤10 pg/ml, HIV^+^_sup_ did not affect the MTT assay unless cells were co-exposed to morphine, indicating a significant synergistic effect (Fig. 1). At p24 concentrations ≥25 pg/ml, there were no significant interactions of HIV^+^_sup_ with morphine. The MTT assay is a measure of the activity of NADH and NADPH-dependent cellular oxidoreductase enzymes [40]–[42]. Although it is frequently used as an indicator of cell survival/toxicity or proliferation, it is only an indirect measure. Therefore, neuron survival was directly assayed using time-lapse imaging (Fig. 2). Like the MTT assay, time-lapse image analysis showed a p24 concentration-dependent decrease in neuron survival over a 48 h period (Fig. 2B). In contrast to the MTT assay, time-lapse image analysis also revealed interactive effects between HIV^+^_sup_ and morphine on cell death at p24 concentrations of 10 and 25 pg/ml. As a p24 concentration of 25 pg/ml caused significant death/toxicity and also showed interactive effects with morphine, this titer was used in all further experiments. Importantly, 25 pg/ml falls within the range of p24 levels detected in the CSF of HAND patients on antiretroviral therapy (p24 = 43.2±16.8 pg/ml) [43].

### Toxic effects of HIV ± morphine in neuron cultures

**Figure 3:**
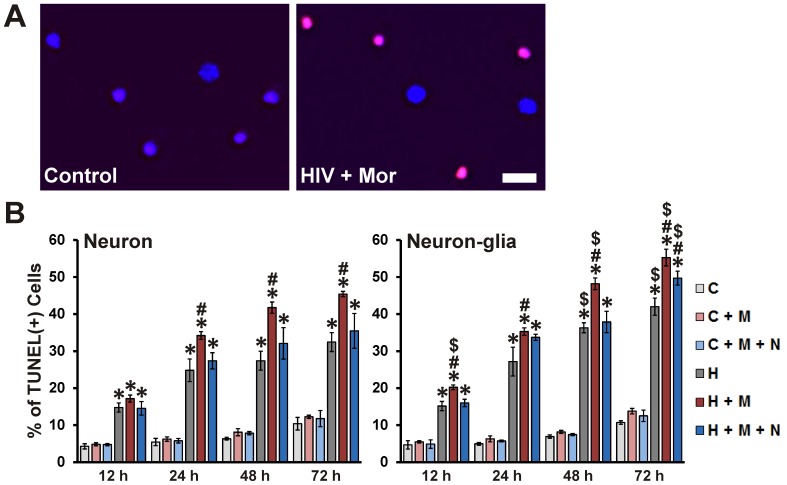
Neuronal apoptosis induced by HIV^+^_sup_ ± morphine. Cells were fixed at specific intervals after treatment and labeled for Hoechst 33342 (blue) and TUNEL (red). (**A**) Digital images of neuronal cultures at 72 h after treatment; scale bar  = 40 µm. (**B**) Apoptosis was assessed by manually counting the percentage of TUNEL(+) cells. Findings were reported as the average percentage of TUNEL(+) cells ± SEM. Significance was analyzed by one-way ANOVA and Duncan's post hoc test, from *n* = 4 separate experiments. At all assessed time points, in both culture systems, all groups exposed to HIV^+^_sup_ showed significantly enhanced neuronal apoptosis (*p<0.05 vs. respective C group). In all cases, except at 12 h in cultures with neurons alone, morphine significantly augmented HIV^+^_sup_-mediated neuronal apoptosis (^#^ *p*<0.05 vs. respective H group). In all cases, except for 72 h in neuron-glia cultures, the interactive effects of morphine were significantly attenuated by naloxone. In most cases, the presence of glia significantly enhanced HIV^+^_sup_ ± morphine-mediated neuron apoptosis (^$^ *p*<0.05 vs. corresponding treatment in neuron cultures; compare panels). C =  Control_sup_; H =  HIV^+^_sup_ (p24 = 25 pg/ml); M =  morphine sulfate (500 nM); N =  naloxone (1.5 µM).

**Figure 4:**
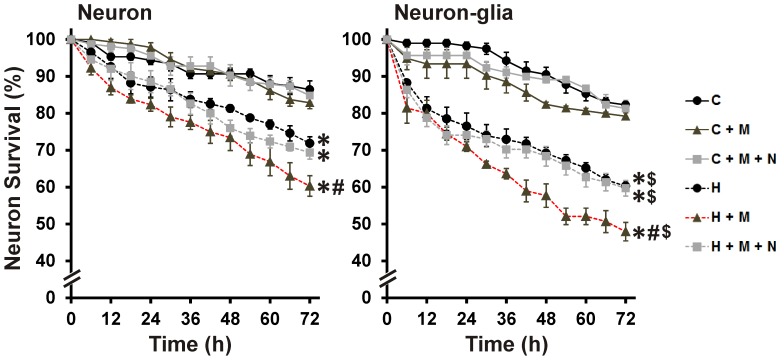
HIV^+^_sup_ ± morphine-mediated neuronal death. Neurons were repeatedly imaged for 72-treatment neuron count ± SEM. Significance was analyzed by repeated measures ANOVA and Duncan's post hoc test, from *n* = 6 separate experiments. Over the period of 72 h, in both culture systems, all groups exposed to HIV^+^_sup_ showed significantly reduced neuronal survival (*p<0.05 vs. C). Morphine significantly enhanced HIV^+^_sup_-mediated neuronal death (^#^ *p*<0.05 vs. H), and the interactive effects of morphine were blocked by naloxone. In the presence of glia, HIV^+^_sup_ ± morphine-mediated neuronal death was significantly enhanced (^$^ *p*<0.05 vs. corresponding treatment in neuronal cultures; compare panels). C =  Control_sup_; H =  HIV^+^_sup_ (p24 = 25 pg/ml); M =  morphine sulfate (500 nM); N =  naloxone (1.5 µM).

Neuronal apoptosis was assessed using TUNEL staining (Fig. 3); all HIV^+^_sup_ treatment groups showed significantly enhanced neuronal apoptosis at all assessed time-points (Fig. 3B; Neuron panel). At all time-points, except 12 h, morphine significantly enhanced HIV^+^_sup_-mediated neuronal apoptosis and the interactive effects of morphine were blocked by naloxone. TUNEL staining is specific for death involving apoptotic pathways, and may not detect all dying neurons. Additionally, TUNEL does not distinguish and permit the exclusion of cells that were dead at the start of the treatment. Time-lapse imaging was used to more exactly follow cell survival/death (Fig. 4). Over the period of 72 h, HIV^+^_sup_ ± morphine treatments significantly reduced neuronal survival in cultures without glia (Fig. 4; Neuron panel). Morphine significantly enhanced neuronal death mediated by HIV^+^_sup_, and interactive effects of morphine were blocked by naloxone.

**Figure 5:**
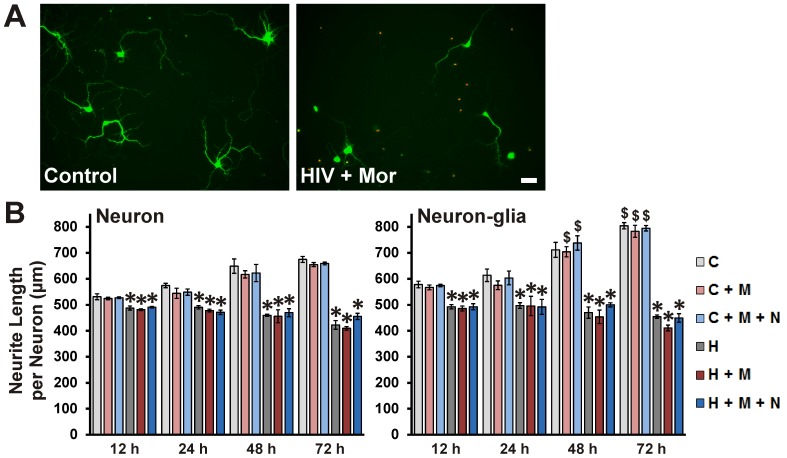
HIV^+^_sup_ ± morphine-mediated neurite damage. Cells were fixed at specific intervals after treatment and labeled for MAP-2 (green) and TUNEL (red). (**A**) Digital images of neuronal cultures at 72 h after treatment; scale bar  = 40 µm. (**B**) The ‘Sholl score’ was assessed only for TUNEL(-) neurons in the digital images and converted into neurite length in µm via a micrometer-scale calibration. The findings were reported as average total neurite length per neuron (µm) ± SEM. Significance was analyzed by one-way ANOVA and Duncan's post hoc test from *n* = 4 separate experiments. At all time-points and in both culture systems, all groups exposed to HIV^+^_sup_ showed significantly reduced neurite length (*p<0.05 vs. C). Morphine did not show a significant interaction with HIV^+^_sup_ treatment. The presence of glia did not have a significant effect on HIV^+^_sup_ ± morphine-mediated neurite damage, but in the presence of glia, Control_sup_-treated groups showed significantly longer neurite length (^$^ *p*<0.05 vs. corresponding treatment in neuron cultures; compare panels). C =  Control_sup_; H =  HIV^+^_sup_ (p24 = 25 pg/ml); M =  morphine sulfate (500 nM); N =  naloxone (1.5 µM).

Sublethal synaptic losses and neuritic pruning are thought to be a major substrate of neurocognitive disorders [4], [44]–[47]. Therefore, effects of HIV^+^_sup_ ± morphine treatment on neuritic arborization were assessed using MAP-2-immunostaining followed by modified Sholl analysis (Fig. 5); only those neurons determined to be alive by TUNEL assay were used in the analysis. At all assessed time-points, HIV^+^_sup_ ± morphine treatment groups showed significantly reduced neurite length (Fig. 5B; Neuron panel). Morphine did not show a significant interaction with HIV^+^_sup_ at any time.

### Role of glia in HIV ± morphine-mediated neurotoxicity

HIV does not infect mature neurons; instead, virotoxins can cause indirect neuron damage via inducing an inflammatory response in activated and/or infected glia [2], [4]–[7]. To determine the role of glia in HIV^+^_sup_ ± morphine-mediated neurotoxicity, treatments were carried out either in the presence or absence of glia. The presence of glia significantly increased the proportion of HIV^+^_sup_ ± morphine-induced TUNEL(+) neurons (Fig. 3B; compare panels). At the earliest time point examined (12 h), HIV^+^_sup_ and morphine displayed a significant interaction; however this only occurred in the presence of glia. Thus, glia appeared to accelerate the HIV^+^_sup_-morphine interaction. At all time points except 72 h, the interactive effects of morphine were significantly attenuated by naloxone. Chronic exposure to naloxone is occasionally ineffective, even when acute blockade reverses morphine effects [48]. This may be due to blocking the cellular effects of the multiple opioids normally released by glia [49], [50]. The presence of glia significantly enhanced HIV^+^_sup_ ± morphine-mediated neuron death over the entire 72 h experimental period (Fig. 4; compare panels).

In the subpopulation of neurons that survived, HIV^+^_sup_ induced significant neurite pruning or growth arrest. The presence of glia did not significantly affect HIV^+^_sup_-induced neurite pruning, even when neurons were co-exposed to morphine. In fact, in the presence of glia, Control_sup_-treated groups had significantly longer neurites (Fig. 5B; compare panels).

### Reversibility of HIV ± morphine-mediated neurite damage

**Figure 6:**
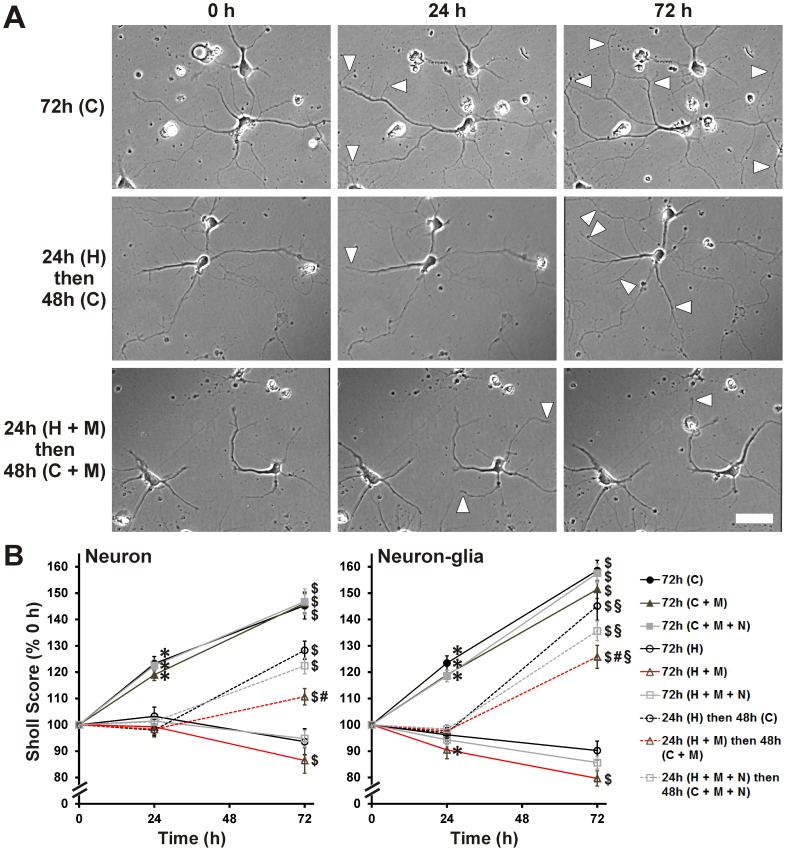
Reversibility of HIV^+^_sup_ ± morphine-mediated neurite damage. Images of pre-selected neurons were captured for 24 h after initial treatments, and for an additional 48 h after treatments were changed as described in Table 1. (**A**) Digital images of neuronal cultures after specified time and treatments (white arrowheads indicate area of neurite outgrowth since previous image); scale bar  = 40 µm. (**B**) Neurons that remained alive until the experiment end (72 h) were assessed for their arborization in images taken at 0, 24 and 72 h, using Sholl analysis. The findings were reported as average Sholl scores at each time, normalized to pre-treatment (0 h) scores ± SEM. Significance was analyzed by repeated measures ANOVA and Duncan's post hoc test, from *n* = 45–60 neurons per treatment group (sampled from 3 separate experiments; at least 15 neurons per group per experiment). Over the period of 24 h, and in both culture systems, HIV^+^_sup_ ± morphine treatments induced neurite growth arrest; in neuron-glia co-cultures, HIV^+^_sup_ + morphine treatment appeared to cause neurite pruning (*p<0.05 vs. 0 h, for corresponding treatment). After removing HIV^+^_sup_ at 24 h, neurite growth arrest was reversible (^$^ *p*<0.05 vs. 24 h, for corresponding treatment); however, if HIV^+^_sup_ ± morphine treatments were continued for 72 h, then neurite growth arrest was persisted. If morphine treatment continued after the removal of HIV^+^_sup_, neurite outgrowth was significantly reduced/delayed compared to neurons returned to Control_sup_ (^#^ *p*<0.05 vs. ‘24 h (H) then 48 h (C)’). This effect of morphine was blocked by naloxone. In the presence of glia, neurite outgrowth after removal of HIV^+^_sup_ was significantly enhanced, even in the continued presence of morphine (^§^ *p*<0.05 vs. corresponding treatment and time point in neuronal cultures; compare panels). C =  Control_sup_; H =  HIV^+^_sup_ (p24 = 25 pg/ml); M =  morphine sulfate (500 nM); N =  naloxone (1.5 µM).

Neurons appear to recover from certain types of sublethal damage caused by HIV-related insults [4], [44], [46]. Therefore, the reversibility of HIV- and morphine-mediated neurite damage was tested. HIV^+^_sup_ ± morphine treatments caused significant neurite growth arrest over the period of 24 h in cultures without glia (Fig. 6B; Neuron panel). With continuous exposure to HIV^+^_sup_ ± morphine for 72 h, neurite length remained significantly reduced. When HIV^+^_sup_ was removed at 24 h, neurites resumed their growth. Interestingly, sustained exposure to morphine by itself was sufficient to reduce and/or delay neurite recovery/outgrowth despite the removal of HIV^+^_sup_. This effect of morphine was blocked by naloxone.

Since glia support neurite outgrowth and synapse remodeling through multiple mechanisms [51]–[54] we tested whether glia play a role in the reversibility of HIV- and morphine-mediated neurite pruning/growth arrest. As in the neuron-only cultures, HIV^+^_sup_ ± morphine treatments caused significant neurite growth arrest over 24 h when glia were present (Fig. 6B; Neuron-glia panel). In the presence of glia, neurite outgrowth was significantly faster after removal of HIV^+^_sup_ than it was in neuron-only cultures (Fig. 6B; compare panels).

### HIV- and morphine-mediated effects on secretion of growth factors and cytokines by glia

**Figure 7:**
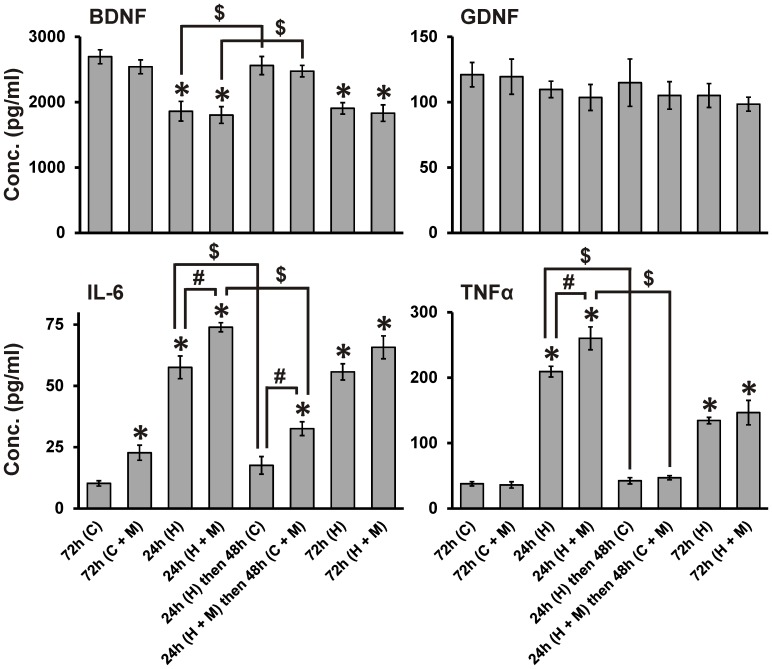
HIV^+^_sup_ ± morphine-mediated effects on secretion of growth factors and cytokines by glia. After specified times and treatments, conditioned medium from mixed glial cultures was collected and assessed for levels of BDNF, GDNF, IL-6 and TNFα by ELISA; Growth factor/cytokine levels were determined based on a standard curve. The findings were reported as average concentrations (pg/ml) ± SEM. Significance was analyzed using a one-way ANOVA and Duncan's post hoc test, from *n* = 3 separate experiments. **BDNF:** HIV^+^_sup_ ± morphine treatments significantly reduced levels of BDNF (*p<0.05 vs. ‘72 h (C)’); after removal of HIV^+^_sup_, BDNF returned to control levels (^$^ *p*<0.05). **GDNF:** HIV^+^_sup_ ± morphine treatments did not have significant effects on GDNF levels. **IL-6:** HIV^+^_sup_ treatment significantly enhanced levels of IL-6; morphine treatment alone also significantly elevated IL-6 levels (*p<0.05 vs. ‘72 h (C)’), and morphine co-treatment significantly augmented HIV^+^_sup_-mediated effects (^#^ *p*<0.05). After removal of HIV^+^_sup_, IL-6 returned to control levels (^$^ *p*<0.05); in the continuous presence of morphine, IL-6 remained at a significantly higher level than control (*p<0.05) and [24 h (H) then 48 h (C)]-treatment group (^#^ *p*<0.05). **TNFα:** HIV^+^_sup_ treatment significantly enhanced levels of TNFα (*p<0.05 vs. ‘72 h (C)’); morphine co-treatment significantly enhanced the HIV^+^_sup_-mediated effect (^#^ *p*<0.05). After removal of HIV^+^_sup_, TNFα levels returned to control values (^$^ *p*<0.05). C =  Control_sup_; H =  HIV^+^_sup_ (p24 = 25 pg/ml); M =  morphine sulfate (500 nM).

Our results show that glial effects on neuron injury and recovery are dependent on the context of HIV and morphine. Glia enhanced HIV-driven neuronal death (Fig. 3B and 4), but accelerated neurite recovery after removal of HIV (Fig. 6B). To determine how glia might direct these outcomes, we examined whether HIV and morphine affect glial production of secreted factors known to influence neuronal survival and outgrowth. ELISA was used to assay levels of the neurotrophic factors (BDNF and GDNF), as well as cytokines (IL-6 and TNFα) that indicate glial inflammatory activation (Fig. 7); they showed multiple response patterns. BDNF levels were significantly reduced by HIV^+^_sup_ ± morphine treatments. BDNF recovered to control levels after removal of HIV^+^_sup_, even though morphine remained present. GDNF levels were unaffected by any treatment. IL-6 levels were increased by HIV^+^_sup_ or morphine treatment alone, and in addition, morphine significantly augmented the effect of HIV^+^_sup_. Although IL-6 levels returned to control after removal of HIV^+^_sup_, the elevated levels were maintained in the continued presence of morphine. TNFα release was significantly increased by HIV^+^_sup_ alone, but not by morphine alone, although morphine co-treatment augmented the effect of HIV^+^_sup_. TNFα levels returned to control after removal of HIV^+^_sup_, even in the continuous presence of morphine. Thus, among the secreted factors whose levels were influenced by HIV and morphine, BDNF, TNFα and IL-6 responded to HIV^+^_sup_ alone, while only IL-6 was affected by morphine itself. Both TNFα and IL-6 showed HIV-morphine interactive effects. Only IL-6 continued to respond to morphine exposure after HIV removal.

### GSK3β as a point of convergence for HIV and morphine

**Figure 8:**
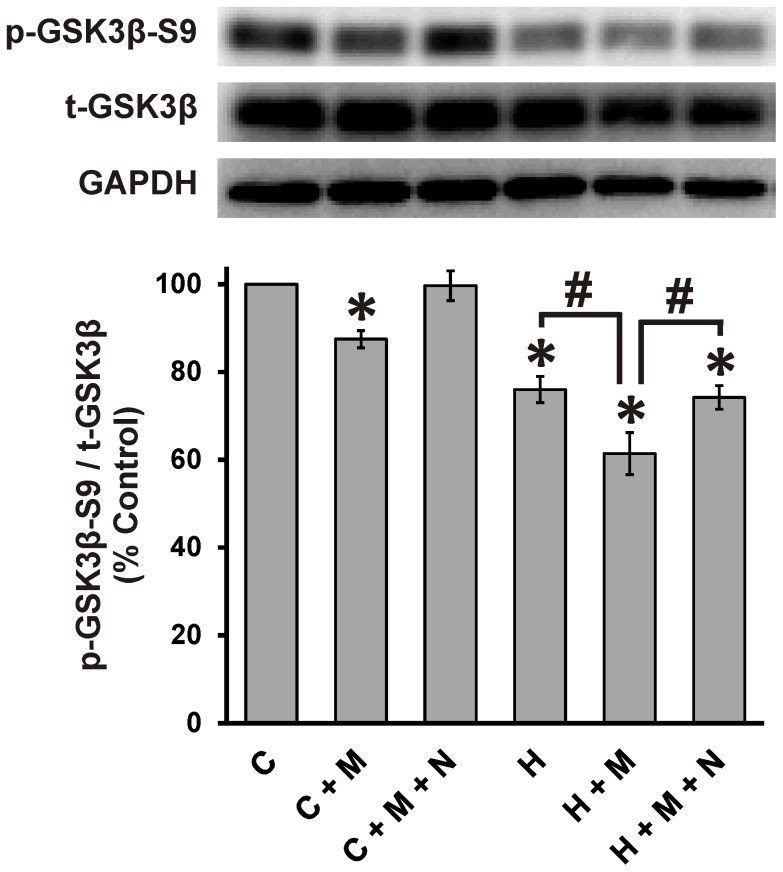
HIV^+^_sup_ ± morphine-mediated GSK3β activation. Cells were lysed and immunoblotted for p-GSKβ-S9 (an inactive form of GSKβ), t- GSKβ (total GSKβ) and GAPDH in neuronal cultures at 24 h after treatment. Findings were reported as a percent of control values of p-GSKβ-S9 levels normalized with t-GSKβ (p-GSKβ-S9/t-GSKβ) ± SEM. Significance was analyzed using a one-way ANOVA and Duncan's post hoc test, from *n* = 3 separate experiments. HIV^+^_sup_ caused significant loss of p-GSKβ-S9 (*p<0.05 vs. C). Treatment with morphine alone also caused significant loss of p-GSKβ-S9, and morphine co-treatment significantly augmented the HIV^+^_sup_-mediated effect (^#^ *p*<0.05). The effects of morphine were blocked by naloxone. C =  Control_sup_; H =  HIV^+^_sup_ (p24 = 25 pg/ml); M =  morphine sulfate (500 nM); N =  naloxone (1.5 µM).

Previous studies have shown that HIV-1 induces neurotoxic effects by enhanced activation of GSK3β [55]–[60], and that GSK3β is also linked to neuropathology seen with opiate-abusing patients [61], [62]. We therefore tested whether GSK3β might be a site of HIV and morphine interactions. Neurons grown in isolation were lysed at 24 h after treatments with HIV^+^_sup_ ± morphine and immunoblotted for phospho-GSK3β-Ser9 (p-GSK3β-S9; an inactive form of GSK3β [63]–[66]), GSK3β (total GSK3β; t-GSK3β) and GAPDH (Fig. 8). HIV^+^_sup_ and morphine by themselves induced significant reduction in p-GSK3β-S9 with respect to t- GSK3β. Morphine co-treatment significantly augmented HIV^+^_sup_-mediated effects. All of the effects of morphine were blocked by naloxone.

## Discussion

Our studies conclusively show that opiates can directly exacerbate the deleterious effects of HIV-1 on neurons in an infective model *in vitro*, although past studies have demonstrated that morphine interacts with the HIV-1 proteins Tat [21], [23] and gp120 [24]. The present studies also confirm and extend prior findings of glial involvement in interactions between opiates and HIV proteins, demonstrating that combined morphine and HIV-1_SF162_ neurotoxicity can be amplified in the presence of glia. Lastly, we found that continuous morphine exposure significantly restricted the ability of neurons to recover from exposure to HIV^+^_sup_. This suggests that HIV-opiate co-exposure may trigger maladaptive cellular responses that persist in the presence of opiates alone, even after HIV infection is mitigated. Importantly, this situation is relevant to opiate-exposed patients whose HIV infection is controlled with cART.

### Experimental models for HIV ± opiate-mediated neurotoxicity

Since HIV is a human-specific disease, models in other species have deficiencies as well as strengths. For example, non-human primates have been an invaluable model to assess interactive effects of the HIV-like simian immunodeficiency virus (SIV) and opiates [67], [68]. However, limited availability and the lack of established simian culture models make mechanistic studies difficult. There are rodent in vivo models that closely mimic viral infection, including an HIV-1 transgenic rat that expresses a majority of HIV-1 proteins without viral replication [69], and “humanized” SCID mice in which establishment of a human immune system in mice permits HIV infection [70]. However, in both cases the peripheral and central target cells are those of the rodent host. Our past *in vivo* studies have used a Tat transgenic mouse in which Tat production is largely restricted to the CNS [20], [25], and we have also examined effects of HIV-1 proteins on murine cells *in vitro* [21], [23], [24], [35], [71], [72]. In general, the findings in culture have paralleled outcomes *in vivo*; all have closely modeled key aspects of neurodegeneration and inflammatory biomarker production seen clinically in the CNS. We are specifically interested in effects on striatal neurons, since the striatum is a major target of HIV-1, and since levels of opioid receptors in the striatum are relatively high [21], [73], [74]. Although primary human cells may be preferable as an *in vitro* model, we have used murine targets since human neurons/glia from specific brain regions are not consistently available, and outcomes frequently show regional specificity [75], [76]. Additionally, murine cultures (a) eliminate human genetic variability in terms of MOR [77], CCR5 [78], [79], and other factors that influence infective and neurodegenerative processes; and, (b) are free from any confounding effects of morphine on HIV replication in human microglia [34]. Still, the issue of species mixing must be considered when interpreting results in this model.

### Neurotoxicity induced by HIV ± morphine

The extent to which opiates contribute to the progression of HAND in the era of cART remains controversial, although some large clinical studies now support moderate interactive effects [27], [29]. Opiate drugs of abuse have been shown to enhance particular damaging effects of HIV-1 proteins *in vitro* [21]–[24], [72]. However, the CNS of HIV-1-infected patients is exposed to a great many other cellular and viral factors released from infected and/or activated cells. Current studies therefore used supernatant from HIV-infected cells to more fully represent the variety of those toxic and protective elements. HIV^+^_sup_ caused neuronal death in a concentration-dependent manner over a range of p24 levels (10–500 pg/ml, Fig. 1 and 2), but significant morphine interactions were observed only at lower p24 levels (10 and 25 pg/ml). Very high levels of neuronal death at p24≥100 pg/ml may have masked interactive effects. If, as our data suggest, HIV-1-opiate interactions are partly governed by the level of infection, HIV-1 patients receiving cART may be especially vulnerable to opiate interactions since cART has greatly reduced the viral load [8]–[10]. The sensitivity of HIV-opiate interactions to levels of infection may also explain some controversy concerning the role of opiates in severity of HAND.

Since synaptic losses and neuritic pruning/degeneration are thought to be the principal substrate underlying HAND [4], [44]–[47], we also examined length of neurites in cells that survived treatments. Our results show that HIV^+^_sup_ reduced the length of neurites, but unlike the cell death results, there were no significant morphine interactions (Fig. 5). Since HIV^+^_sup_ and morphine can induce multiple pathways, it is easily envisioned that interactions may differ between outcome measures. In some instances, cumulative reductions in synapses and dendritic simplification may culminate in cell death. Alternatively, neurite pruning may result in significant loss of cellular functions, but neurons may remain alive [71], [80]. Control treated groups actually showed an increase in the length of neurites over the same timeframe. This suggests that neurite length changes mainly reflected neurite growth arrest/inhibition. Results from repeated neurite length assessments of individual cells (Fig. 6) support this hypothesis. These conclusions are in conflict with some previous studies [71], [72], [81], where reduction in neurite length was mainly attributed to pruning of existing neurites. Disparate findings may reflect different types of neurons, their age and relative maturity, the response of neurons to individual viral proteins versus the multiple stimuli in HIV^+^_sup_, and the selection criteria for neurons; we specifically evaluated sub-lethal neurite length changes by assessing only [TUNEL(-)] cells instead of the entire population.

Although many experimental and epidemiological studies have indicated a link between opiate drug exposure and HAND severity, the mechanisms underlying interactions between HIV-1 and opiates remain largely obscure. HIV-1 is known to induce neurotoxic effects through abnormal activation of GSK3β, and the GSK3β inhibitors, lithium (Li) and sodium valproate (VPA), ameliorate HIV-1-mediated neurotoxicity [55]–[60]. GSK3β signaling is also implicated in neuropathologic responses to opiates. For example, the accelerated deposition of hyperphosphorylated tau that occurs in the CNS of young opiate abusers [61], [62] may be related to elevated GSK3β expression seen in opiate abusers [61] since GSK3β is a principal tau kinase [82]–[84]. GSK3β plays a crucial role in regulating the levels and function of various structural and signaling proteins in neurons including tau, MAP2, β-catenin, activator protein 1 (AP-1), cyclic AMP response element binding protein (CREB), heat shock factor-1 (HSF-1), and among others, all of which regulate neuronal plasticity, gene expression and survival [65], [66], [85]. GSK3β is thus well-positioned to be a potential convergence point for interactions between HIV-1 and opiates that regulate neuronal damage. Our results show that morphine co-exposure significantly augments HIV^+^_sup_-mediated GSK3β-activation (Fig. 8), supporting this hypothesis.

### Role of glia in HIV ± morphine-mediated neurotoxicity

Opiates exacerbate the release of numerous factors with neurotoxic potential from glia exposed to HIV [16], [26], [86], and alone or in concert with HIV can disrupt certain neuron-supportive functions of glia, including glutamate buffering, free radical scavenging, phagocytosis and release of neurotrophic factors [23], [86]–[88]. It is easily appreciated that glia might play a crucial role in HIV-opiate interactions; in our previous studies glia were actually required for interactive neurotoxicity between morphine and HIV-1 Tat [23]. In the present study, morphine significantly enhanced HIV^+^_sup_-mediated striatal neuron death even in the absence of glia. One obvious interpretation is that morphine interacts with factors in addition to HIV-1 Tat in the HIV^+^_sup_. Even among R5 strains, unique gp120 sequences may result in a different degree of interaction between opiates and HIV [24]. While glia are clearly important determinants of neurotoxic HIV-opiate interactions, some interactions, perhaps those involving factors other than Tat, seem to occur directly upon neurons.

Glia also modified neurite recovery, enhancing outgrowth when HIV was removed. The effect of glia on neurons is never entirely positive or negative but instead reflects the net input of various effectors that either promote or damage neurite/neuron structure and function [51]–[54]. In this context, our finding that glial production of BDNF is suppressed by HIV^+^_sup_ but then rebounds to control levels after removal of HIV^+^_sup_ shows a return towards a more trophic glial function. The normalization of proinflammatory cytokines TNFα and IL-6 after HIV removal indicates a similar trend, although note that continued exposure to morphine partly abrogates the effect of removing HIV (Fig. 7).

Overall, our results show that cellular and viral products released from HIV-1_SF162_-infected leukemic monocytes have significant negative consequences on striatal neurons. Coincident exposure to morphine worsens neuronal outcomes in a concentration- and time-dependent manner. This is especially true when glia are present, although the net effects of glial exposure depend upon the local levels of virus and opiates. At lower viral titers, HIV^+^_sup_ has sublethal effects on growth of neurite arbors, indicating that neurons may undergo functional changes long before they die. This may be quite relevant to the situation in HIV-infected patients where dendritic/synaptic plasticity, not neuron death, is the presumed substrate of HAND. Diminished infection levels in the CNS are probably critical in reversing HIV-driven neurite damage, although our results caution that chronic exposure to opiates may inflict damage even in the absence of HIV.
